# Combination of Membrane-Based Pre-Treatment Techniques and Heterogeneous Photocatalysis to Obtain High-Quality Effluents from Produced Water

**DOI:** 10.3390/molecules30122532

**Published:** 2025-06-10

**Authors:** Greta Brocchetto, Iván Sciscenko, Marco Minella, Lorenzo Craveri, Erica Bertozzi, Marco Malaguti, Marco Coha, Alberto Tiraferri, Davide Vione

**Affiliations:** 1 Department of Chemistry, University of Turin, Via Pietro Giuria 5, 10125 Turin, Italy; greta.brocchetto@unito.it (G.B.); ivanmatias.sciscenko@unito.it (I.S.); marco.minella@unito.it (M.M.); 2 Department of Environment, Land and Infrastructure Engineering (DIATI), Politecnico di Torino, Corso Duca Degli Abruzzi 24, 10129 Turin, Italy; lorenzo.craveri@polito.it (L.C.); erica.bertozzi@polito.it (E.B.); marco.malaguti@polito.it (M.M.); marco.coha@polito.it (M.C.)

**Keywords:** advanced oxidation processes, produced water, oil and gas extraction, treatment trains, water treatment

## Abstract

Produced water is the waste aqueous phase from petroleum extraction. As it contains salts, a high organic load, and toxic organic compounds, it should be treated before disposal or reuse. In this research, the combination of membrane processes (microfiltration or membrane distillation) with TiO_2_-based heterogeneous photocatalysis was assessed to treat synthetic produced water. Pre-treatment with both microfiltration and membrane distillation removed the majority (90–98%) of large organic compounds (humic acids) from produced water. Moreover, membrane distillation also eliminated salt (sodium chloride). However, membrane processes only removed 10–50% of phenol, used here as proxy for low-molecular-weight toxic organic compounds. For this reason, membrane permeates, from microfiltration and membrane distillation, underwent a further photocatalytic treatment aimed at phenol degradation. The application of TiO_2_ photocatalysis to membrane distillation permeates was successful (100% phenol removal in 5 min), while the high chloride concentration of microfiltration permeates acted as inhibitor of the photocatalytic process. Overall, good-quality water may be obtained from the combination of membrane distillation and heterogeneous photocatalysis, which performed much better than the two techniques used separately. Indeed, while membrane distillation was not able to remove phenol, produced water was too complex a matrix to be effectively treated with TiO_2_/UV photocatalysis alone.

## 1. Introduction

Nowadays, oil and gas (O&G) extraction still represents the most substantial player in the energy sector, with oil consumption reaching 94 million barrels per day in 2022 [1,2]. Among the critical issues that derive from O&G extraction and use, water consumption is one of the most pressing, given that the production of wastewater, commonly referred to as “produced water” (PW), accounts for 80% of the total waste from the extraction activities. Daily PW production is estimated to be around 250 million barrels. PW derives from the injection of large volumes of water into the subsoil during O&G extraction, to achieve high resource recovery by counteracting the subsoil pressure. Water thus resurfaces containing a great variety of compounds, such as metals, salts, and organics, including a range of toxic aromatic molecules [3]. Consequently, efficient decontamination of PW is gaining interest due to increasingly stringent regulations, as well as challenges and costs of PW management. Indeed, tackling the safe discharge of treated PW and proposing sustainable and effective PW management solutions is of crucial importance for the safety of humans and the environment.

In general terms, water treatment units designed to decontaminate PW streams consist of a primary treatment to separate oil and water, an optional secondary treatment unit designed to remove suspended substances and to increase oil removal, and a final tertiary treatment unit (polishing train) [4,5]. While the first two treatment steps are sufficiently standardized and effective, a variety of polishing technologies are available as potential candidates to reduce the O&G content below legal limits, and to achieve product water quality suitable for safe discharge or beneficial reuse/recycling [4]. The most widely used polishing solutions substantially differ in their working principles, but among the most recently investigated and promising techniques, membrane-based separation processes and advanced oxidation processes (AOPs) play a crucial and, interestingly, complementary role [6].

Regardless of their order in the polishing train, membrane separation techniques such as microfiltration (MF) and membrane distillation (MD) show their advantages in terms of compactness, flexibility, and modularity. While MF is a pressure-driven process based on a size exclusion mechanism, MD is a thermal-driven technique that exploits a vapor pressure gradient to obtain a vapor flux across a hydrophobic membrane. The main difference in terms of separation performance when treating a PW stream is the capability of MD to remove dissolved ions and other non-volatile constituents, while MF effectiveness is closely related to the membrane pore size, thereby narrowing the range of compounds that can be removed. MD becomes highly interesting in all cases where the influent stream has a total dissolved solids concentration above roughly 50 g L^−1^, a freshwater product is desired, and especially when abundant waste heat is available—a condition that is actually often met in extraction installations. However, a significant spectrum of compounds cannot be removed by the aforementioned processes, most notably volatile components and low-molecular-weight organics, which are virtually always present in PW and are often toxic [7]. For this reason, AOPs such as heterogeneous photocatalysis can significantly increase the decontamination performance of PW streams.

AOPs are processes in which extremely reactive species (e.g., hydroxyl radicals, HO^•^) are produced to degrade (bio)recalcitrant compounds [8]. In the case of heterogeneous photocatalysis, these reactive species are generated by irradiating a semiconductor with light of a certain wavelength (λ ≤ band gap energy, E_g_), which causes an electronic transition from the valence band to the conduction band. The generated electron in the conduction band can induce reductive processes (e.g., the reduction of dissolved oxygen into superoxide radical, O_2_^•−^), while the hole (h^+^) in the valence band is an oxidant that can cause pollutant oxidation directly and/or through ^•^OH (mainly adsorbed at the photocatalyst surface as ^•^OH_(s)_) [9,10]. Titanium dioxide (TiO_2_) is by far the most common photocatalyst employed for water treatment, due to its high chemical stability, relatively high availability, low cost, and environmental compatibility [11]. Considering that E_g_ = 3.2 eV (anatase polymorph), ultraviolet A (UV-A) light (λ < 390 nm) is energetic enough to drive photocatalytic reactions with this material [12]. Because of these properties, the use of TiO_2_ photocatalysis has been previously studied for PW treatment [13,14,15].

Substantial research has been conducted in recent years, focusing on combining a pre-treatment step based on AOPs with a subsequent, polishing step including a membrane-based separation unit, showing that even a partial oxidation can be beneficial for membrane-based PW treatment by increasing productivity and reducing fouling [7,16]. This strategy is particularly effective for influent streams with high fouling potential and a small amount of low-molecular-weight organic compounds. On the other hand, certain influent streams may contain substances that interfere with the performance of AOPs. In these cases, the complexity of the raw PW matrix can lead to competitive reactions and reduce the removal efficiency of organic pollutants [17,18]. Additionally, high wastewater salinity can inhibit TiO_2_-based photocatalysis, resulting in both lower degradation performance and a shorter catalyst lifespan [19,20,21].

A different strategy encompasses the deployment of membrane processes as a pre-treatment to AOPs, which are instead applied as a polishing step. An example is the application of nanofiltration to pre-treat urban wastewater spiked with carbamazepine, which was then removed from the permeate with the Fenton reaction [22]. The Fenton process was highly aided by the removal of interfering agents (mostly large organic molecules) by the membrane. However, PW has several peculiarities that differentiate it from urban wastewater, such as larger occurrence of volatile organic compounds [17].

In this work, MD and MF processes are investigated in laboratory settings as alternative pre-treatments in terms of productivity and effluent quality, starting from simplified, synthetic PWs comprising humic acids, phenol, and varying concentrations of sodium chloride. The water streams from the membrane units are then subjected to heterogeneous catalysis treatment by means of TiO_2_ and UV-A radiation, to produce high-quality water and to unravel the role and the impact of typical PW compounds on the degradation performance.

## 2. Results and Discussion

Synthetic produced waters (PWs) were formulated using humic acid, sodium chloride, and phenol to simulate the typical organic, saline, and toxic components, respectively found in real PW. Four PW solutions with varying salinities (0–1000–10,000–100,000 mg L^−1^ total dissolved solids, TDS) were subjected to membrane-based pre-treatment via membrane distillation (MD) or microfiltration (MF). MD was conducted in direct contact mode using a hydrophobic PTFE membrane at controlled temperatures (feed/distillate: 50/25 °C), while MF employed a TiO_2_-based ceramic membrane under cross-flow at ambient temperature (25 °C). Treated effluents were subsequently processed via TiO_2_ (0.5 g L^−1^) photocatalysis under UV-A irradiation. Degradation of phenol and total organic carbon (TOC) was monitored using high-performance liquid chromatography with ultra-violet detection (HPLC-UV) and TOC analysis, respectively.

### 2.1. Membrane Pre-Treatment of PW: Comparison Between MF and MD

The performance of the investigated membrane-based pre-treatment techniques (MF or MD) was assessed in terms of productivity and reduction of the following PW parameters: TOC (mostly accounted for by humic substances), electrical conductivity (proportional to the residual NaCl concentration), and phenol concentration, the latter being indicative of low-molecular weight toxic organic species in PW.

**Figure 1:**
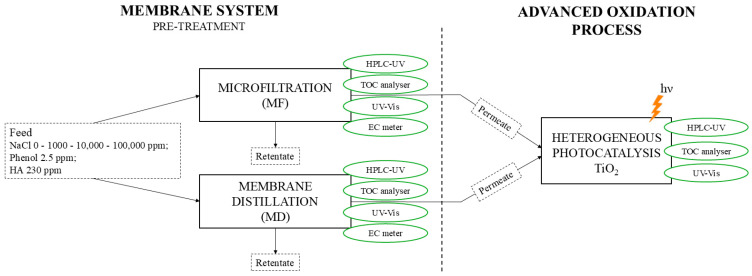
Schematic representation of the experimental setup used in this study. The treatment system consists of two alternative membrane-based pre-treatment processes (microfiltration, MF, or membrane distillation, MD) applied to a synthetic PW feed. The permeate obtained from each membrane process was subjected to the subsequent advanced oxidation process (AOP), which involved heterogeneous photocatalysis using TiO_2_ under UV irradiation. The retentate was not subjected to any treatment. Water quality parameters were monitored at different stages using HPLC-UV, TOC analyser, UV–Vis spectrophotometer, and conductivity meter (EC).

To enhance the clarity of the experimental methodology, a schematic diagram of the overall reaction system is provided in Figure 1. The diagram illustrates the sequential process steps, including the pre-treatments performed via MF and MD, followed by the heterogeneous photocatalysis (TiO_2_) stage. The figure also highlights the chemical analyses performed at each stage of the process.

**Figure 2:**
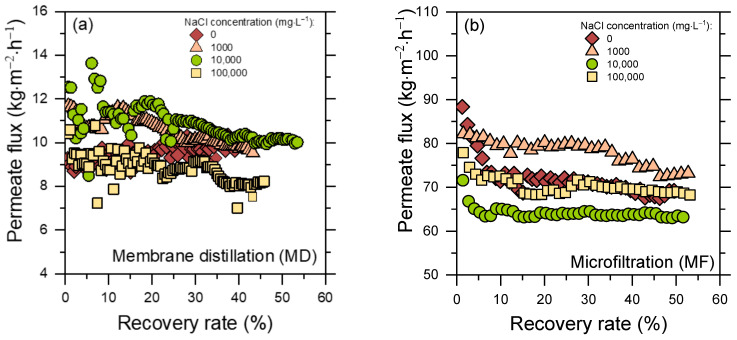
Permeate flux measured with membrane-based pre-treatment filtrations with different synthetic PWs. (**a**) Results of MD tests. (**b**) Results of MF tests. Four NaCl concentrations were investigated: (red diamond) 0 mg L^−1^; (orange triangle) 1000 mg L^−1^; (green circles) 10,000 mg L^−1^; (yellow squares) 100,000 mg L^−1^. Fluxes (kg m^−2^ h^−1^) are reported as a function of recovery rate (%).

Figure 2 presents the experimental permeate flux obtained in MD (1a) and MF (1b) tests with synthetic PWs characterized by different NaCl concentrations (0, 1000, 10,000, and 100,000 mg L^−1^), as a function of the recovery rate. In MD the presence of salts and organic compounds may negatively affect the process, possibly causing fouling (deposition of material on the membrane surface and blocking of pores), wetting, and/or scaling (formation of salt crystals on the membrane) [23,24,25,26,27,28].

The MD permeate flux values were in the range of 8–12 kg m^−2^ h^−1^ for all feed salinities, with <20% differences for the various investigated feed solutions. It is reasonable to expect a slightly lower flux from feed solutions with higher salinity, due to a reduced driving force and the more impactful scaling phenomenon [27,29,30]. Except for the feed with no added salt (PW 0, which showed almost constant values during the experiment), the MD permeate flux decreased over time, likely because of fouling and scaling phenomena. The presence of both organic compounds and salts, which may deposit over time and attach on the membrane surface, would lead to pore blocking and reduced vapor flux [24,25].

Permeate fluxes for MF were evidently higher than those characterizing the MD treatment, and they were in the 60–85 kg m^−2^ h^−1^ range. All four MF experimental curves showed a decreasing trend, with the 0 mg L^−1^ solution displaying the highest drop (around 23%). As in MD, a major role in this behavior could be played by fouling due to the deposition of humic acids, which may lead to pore blockage and cake layer formation [31,32,33]. No evident relationships between NaCl concentration and flux trends were noted, as partly expected due to the fact that the MF membrane would not reject dissolved ions. Furthermore, the ionic strength likely affected humic acids conformation in a way that, however, did not substantially impact the flux.

**Figure 3:**
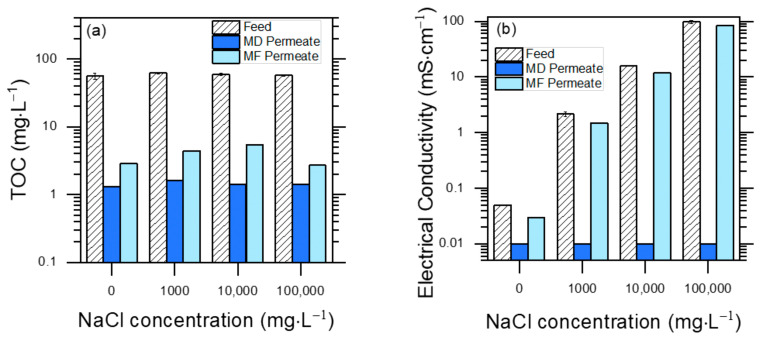
Separation performance measured with membrane-based pre-treatment filtrations, with different synthetic PW solutions. Note the logarithmic scales on both Y axes, due to very high differences in TOC values between feed and permeates, and in electrical conductivity among different feeds. (**a**) TOC values of feed and permeate solutions for both MD and MF treatments. Feed TOC variability is an index of reproducibility of measurements (TOC_o_ = 58 ± 6 mg L^−1^ (μ ± σ)). (**b**) Conductivity of feed and permeate solutions for both MD and MF treatments. Conductivity values varied from 10 to over 100,000 μS cm^−1^.

The effects of MD and MF pre-treatments on the TOC values of PW are displayed in Figure 3a (feed: original PW; permeate: membrane-treated PW) for different initial contents of NaCl. Both techniques were effective at removing humic substances, which accounted for >95% of the initial TOC (the rest was accounted for by phenol). MD performed better than MF, achieving 98–99% TOC removal vs. 95–97%.

These findings suggest that humic acids were hardly able to pass through MF pores due to their molecular size, and they were also not volatile enough to pass through the MD membrane. The compared performance of MD and MF was different in the case of conductivity reduction (Figure 3b).

The MF treatment decreased conductivity by less than 10% under the different conditions, while MD decreased it to near the instrumental detection limits (~0.01 mS cm^−1^). While Na^+^ and Cl^−^ ions are not retained by the MF membrane, their non-volatility ensured an effective rejection by MD. This is an advantage of MD, because in the case of MF pre-treatment, an additional salt elimination pathway would be required to enable PW reuse.

That being said, the increase in NaCl concentration in the feed solution could facilitate in time the development of wetting of the MD membrane, impairing the process [34]. However, no substantial increase in distillate conductivity was noted in this study, suggesting no considerable salt passage and, therefore, no or negligible membrane wetting within the timeframe of our experiments.

**Figure 4:**
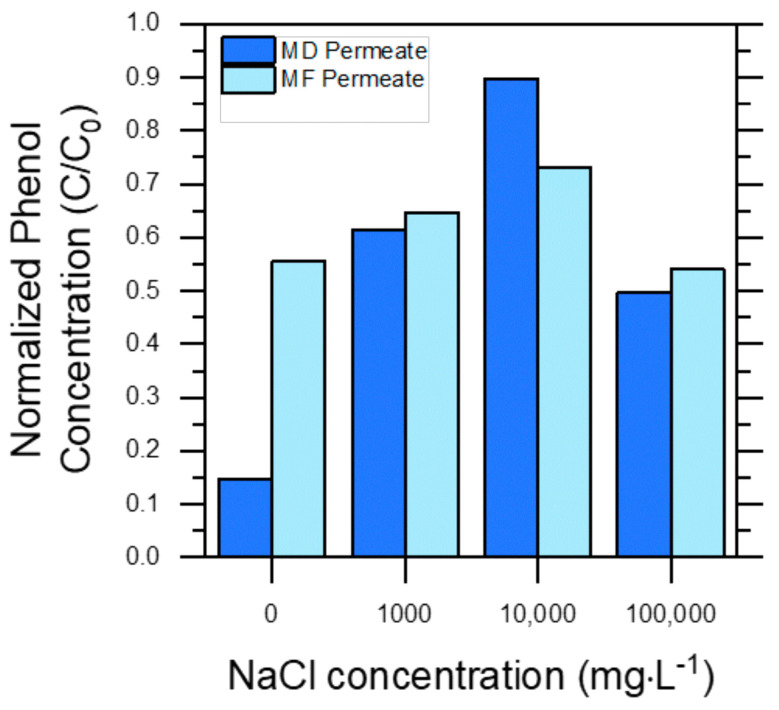
Normalized phenol concentrations (C/C_o_) in the permeate water obtained through MD and MF processes, with respect to their initial feed water (PW) concentration, reported as a function of initial NaCl concentrations. Dark blue bars (left-hand bars) refer to permeate from the MD treatment, while light blue bars (right-hand bars) refer to permeate from the MF treatment.

Finally, Figure 4 shows membrane pre-treatment performance towards the elimination of phenol. Phenol rejection was far from quantitative in either case, and it was always ≤50% in the presence of NaCl. In the case of MF, the membrane pores were too large to remove phenol; thus, any degree of phenol rejection was unexpected. Interestingly, MF even produced a slightly higher phenol removal than MD in the case of PW 10,000. This result could be explained by the fact that phenol can interact with humic acids, especially when the ionic strength of the solution is high [35]. Under such circumstances, phenol–humic acids aggregates could be retained by the MF pores. Differently from PW 0, in the other PWs, the presence of NaCl likely induced a salting-out effect that increased phenol volatility and accounted for quite low phenol rejection by MD in the most saline solutions (e.g., ~10% for PW 10,000).

Overall, these results indicate that, while MF can provide a much higher productivity in terms of permeate flux (in our case, 60–90 vs. 8–14 L m^−2^ h^−1^), it is able to eliminate only large organics from PW, while MD can eliminate both large organics and dissolved ions. However, both techniques failed to effectively reject the most toxic species (phenol) in the presence of NaCl. Membrane-treated PW would thus retain a considerable fraction of its initial toxicity (usually >50%, based on phenol concentration in permeate vs. feed), which means that membrane treatment alone cannot achieve the target for PW discharge or reuse. For this reason, in this work we have studied a combination of MD or MF pre-treatment and TiO_2_-based photocatalysis to achieve the additional goal of the degradation of toxic organic compounds.

### 2.2. Photocatalytic (TiO_2_/UV) Decontamination of Membrane-Treated PW

**Figure 5:**
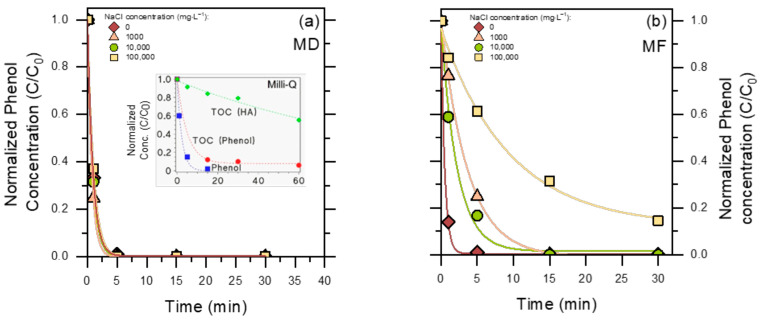
Phenol degradation profiles in the PW permeate, upon UV-A irradiation in the presence of 0.5 g L^−1^ TiO_2_. (**a**) MD permeate; (**b**) MF permeate. The insert in (**a**) shows the time evolutions of 2.5 mg L^−1^ phenol and related TOC in Milli-Q (ultra-pure) water, in the presence of 0.5 g L^−1^ TiO_2_ under UV-A irradiation, as well as of TOC of an irradiated solution containing 0.5 g L^−1^ TiO_2_ + 5 mg L^−1^ HA. The curves represent data fit with an exponential (pseudo-first order) decay function.

The time trends of photocatalytic phenol degradation in MD- and MF-pre-treated PW are reported in Figure 5. Interestingly, irradiated TiO_2_ was able to induce complete phenol disappearance in 5 min in MD-pre-treated PW (Figure 5a), while the process was considerably slower for MF pre-treatment (Figure 5b). By comparison, negligible degradation of phenol was observed with either UV-A irradiation alone, without TiO_2_ (phenol does not absorb UV-A radiation), or in the presence of TiO_2_ in the dark. Indeed, hydrophilic phenol undergoes negligible adsorption on the surface of TiO_2_ [36].

In the case of MF, photodegradation was quite fast in the absence of chloride but slowed down considerably in the Cl^−^-containing matrices, especially as far as 100,000 mg L^−1^ initial NaCl was concerned (more than ten-fold slower phenol degradation in PW 100,000 compared to PW 0).

While it is true that the MF permeate contained more humic substances than the MD permeate (see Figure 3a), the experiments involving both permeates without chloride (Figure 5) suggest that a few mg L^−1^ humic acids would not be the critical factor to slow down phenol degradation in the MF permeate, while chlorides likely were critical. Indeed, chloride is known to inhibit TiO_2_-based photocatalysis by occupying adsorption sites on the photocatalyst surface (not relevant for phenol, which does not significantly adsorb onto TiO_2_ [20,36]) and, more significant for phenol, by scavenging surface ^•^OH_(s)_/h^+^ to yield less reactive species such as Cl^•^/Cl_2_^•−^ [37]. To make a comparison, the second-order reaction rate constants of phenol with Cl_2_^•−^ (2.5 × 10^8^ M^−1^ s^−1^) [38] and Cl^•^ (1.4 × 10^9^ M^−1^ s^−1^) [39] are ~55 and 10 times lower than the corresponding rate constant between phenol and ^•^OH (1.4 × 10^10^ M^−1^ s^−1^), respectively [40]. Moreover, high concentrations of chloride neutralize the coulombic repulsion among the TiO_2_ particles and promote their coagulation, which decreases the exposed surface area and forms points of contact among particles that work as h^+^-e^−^ recombination centers [41]. Therefore, effective chloride elimination by MD is the likely factor to explain why phenol underwent better photocatalytic degradation in the MD permeate than in the MF permeate.

**Figure 6:**
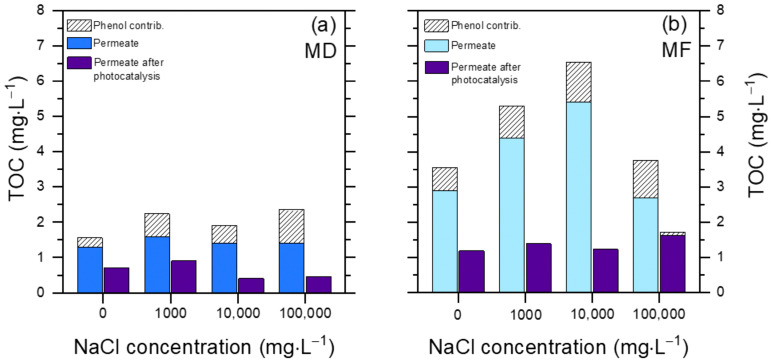
TOC removal from the PW permeate, upon UV-A irradiation in the presence of 0.5 g L^−1^ TiO_2_ after 60 min. (**a**) MD permeate; (**b**) MF permeate. The contribution of phenol to the TOC value of the permeate solution before and after photocatalytic degradation is highlighted (in the latter case, it was the TOC value accounted for by the residual phenol that still occurred in the solution).

Figure 6 reports the results of the TOC decrease after 60 min photocatalytic degradation. The TOC elimination is usually slower than the degradation of the primary substrates ([42], and see also the insert in Figure 5a), thus these experiments where allowed more time (60 min) compared to those dedicated to phenol elimination (30 min).

Two interesting issues are apparent: (i) complete mineralization was not achieved in 1 h in either the MD or the MF permeate, and (ii) compared to MF, the MD permeate showed lower initial and final TOC values.

The TOC value encompasses both phenol and humic acids (HA), and phenol made up a significant fraction of the TOC (15–40%) in both permeates before photocatalytic treatment (Figure 6). This is accounted for by the much better rejection of HA compared to phenol by both MD and MF. Therefore, the phenol/HA ratio was much higher in the permeates compared to the original PW composition. At the same time, phenol was largely or completely degraded after 60 min irradiation in the presence of TiO_2_.

The ability of TiO_2_ to degrade and mineralize phenol alone in ultra-pure (Milli-Q) water is reported in the insert of Figure 5a, showing that complete removal of 2.5 mg L^−1^ phenol and ~90% removal of the corresponding TOC (initial TOC_o_ ~2 mg_C_ L^−1^) could be achieved within 60 min irradiation. On this basis, most of the residual TOC remaining in the MD/MF permeates after photocatalytic treatment (Figure 6) was likely due to HA and not to phenol transformation products. Coherently, photocatalytic degradation of 5 mg L^−1^ HA in ultra-pure water (comparable TOC_o_ as 2.5 mg L^−1^ phenol) gave only ~40% removal of TOC after 60 min (see insert of Figure 5a). The overall data suggest that mineralization of phenol by TiO_2_ is easier and faster compared to the mineralization of HA.

**Figure 7:**
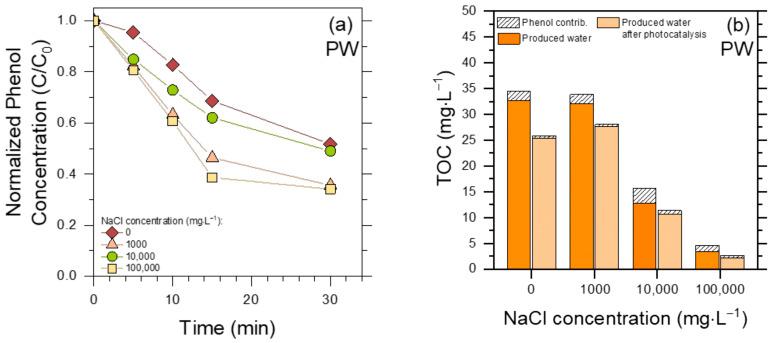
(**a**) Phenol degradation profiles in raw PW at different initial concentrations of NaCl, upon UV-A irradiation in the presence of 0.5 g L^−1^ TiO_2_. (**b**) TOC removal upon the same photocatalytic treatment of PW (but for 60 min treatment time).

The results reported so far indicate that membrane treatments alone are not effective enough in PW decontamination, while the combination of membrane (especially MD) pre-treatment and heterogeneous photocatalysis performs better. The question is still open, whether photocatalysis alone can be appropriate. Figure 7 reports the results obtained by direct photocatalytic treatment of the initial (raw) PW, without the membrane step, regarding both phenol elimination and TOC decrease.

First of all, the photocatalytic degradation of phenol in PW was strongly inhibited in all conditions compared to membrane permeates (compare Figure 7a with Figure 5), even in the absence of chloride. This result suggests that, differently from the case of membrane pre-treatment, the very large amount of humic acids in the original PW was able to inhibit the photocatalytic degradation of phenol. In this framework, humic acids can both scavenge reactive species (^•^OH_(s)_/h^+^, the former being mostly involved in phenol degradation [36]) and compete with TiO_2_ for radiation absorption [43].

A second issue is that, differently from membrane (and especially MF) permeates, photocatalytic degradation in raw PW was interestingly faster in the presence of high NaCl contents. This result, obtained in a rather complex matrix as raw PW, can be accounted for when considering that (i) Cl^•^/Cl_2_^•−^, being less reactive, would be more selective than ^•^OH_(s)_ [38,40], and that (ii) phenol is an easier-to-degrade molecule than average humic acids (insert, Figure 5a). Therefore, if humic acids occur together with chloride, as they do in PW, they will scavenge ^•^OH_(s)_ to a large extent but be less able to scavenge Cl^•^/Cl_2_^•−^. The eventual outcome is that phenol degradation by ^•^OH_(s)_ in raw PW is inhibited by humic acids, but degradation is faster in the presence of high chloride concentrations because Cl^•^/Cl_2_^•−^ react with phenol more selectively than with humic acids.^17^ Another possible effect of chloride is that the scavenging of ^•^OH_(s)_/h^+^ to produce Cl^•^/Cl_2_^•−^ would inhibit recombination between ^•^OH_(s)_/h^+^ and e^−^ [37], resulting in quite high photocatalytic generation of Cl^•^/Cl_2_^•−^, especially at the highest chloride concentrations.

As far as the TOC is concerned (Figure 7b), comparison between the TOC values before and after TiO_2_ irradiation suggests in each case a limited removal (<50%) by photocatalytic treatment. More interestingly, the measured TOC value before photocatalysis decreased with increasing NaCl, and it was particularly low (~5 mg L^−1^) at the highest NaCl concentration. Consider that the investigated PW samples all had the same initial contents of humic substances and phenol, and TiO_2_ was added to them all. However, filtration to remove TiO_2_ (0.45 µm syringe filters) was required before TOC measurement, and the filtration process removed part of the humic acids together with the photocatalyst. This phenomenon would take place to a larger extent at the highest NaCl concentrations, which facilitate aggregation and precipitation of humic acids [44]. Therefore, the addition of TiO_2_ to PW, followed by filtration before TOC measurement, would carry out a physical elimination of humic acids, independently of irradiation, to a larger extent than their photocatalytic degradation. These findings suggest that raw PW is too complex a matrix for TiO_2_-based photocatalysis to be effective as a stand-alone treatment.

### 2.3. Comparative Insights into TiO_2_ Photocatalysis in Saline and Complex Wastewater Matrices

Overall, the pre-treatment via membrane distillation (MD) or microfiltration (MF) followed by TiO_2_ photocatalysis demonstrated satisfactory performances, achieving phenol degradation ratios of up to >99% within just 5 min of treatment, with a minimum of 80% degradation after 30 min of irradiation under the highest concentration of NaCl (100,000 mg L^−1^), in the case of MF pre-treatment. These results are comparable with similar works, highlighting that the use of membrane-based pre-treatment can avoid the use of sophisticated photocatalysts [3,45,46]. The obtained phenol removal rate is also similar to results obtained in low-salinity water matrices, such as drinking water: Valadez-Rentevria et al. (2021) employed CuS/TiO_2_ nanoparticles in drinking water and obtained ~90% degradation of chlorophenol (20 mg L^−1^ initial concentration) after 150 min of irradiation [47]. Andrade et al. (2014) obtained 70% phenol (85 ppm initial concentration) removal after 6 h of UV-visible light exposure, using a composite of TiO_2_ and copper-loaded nanoporous carbon in a carbon matrix [48]. Similar results could be observed for TiO_2_ alternatives, such as ZnO. Vasantharaj et al. (2021) reached ~90% of organic dyes (methylene blue and malachite green, 10 mg L^−1^ initial concentration) degradation after 150 min of sunlight irradiation in wastewater [49]. Lakshmi et al. (2019) reported that La-ZnO-polyacrylonitrile fiber nanocomposites could achieve 98% degradation of atrazine (10 mg L^−1^ initial concentration), a persistent pesticide, in 60 min [50,51].

## 3. Materials and Methods

### 3.1. Produced Water (PW) Preparation

Three distinct synthetic PWs were prepared to both mimic and simplify the typical composition found in real PWs, with the goal of investigating the contribution of individual components of a treatment train aimed at obtaining high-quality water [7,52]. Specifically, humic acid (HA, Alfa Aesar, Karlsruhe, Germany) and sodium chloride (NaCl, Carlo Erba, Cornaredo, Italy) were used to mimic, respectively, total organic carbon (TOC) and total dissolved solids (TDS) at varying concentrations (TDS = 1000 mg L^−1^, 10,000 mg L^−1^, or 100,000 mg L^−1^). Additionally, phenol (≥99%, Chem-lab NV, Zedelgem, Belgium) was added at a concentration of 2.5 mg L^−1^ across all solutions, as a representative substance for the toxic aromatic compounds occurring in produced waters that often reach mg L^−1^ levels [5]. Additionally, a solution with the same TOC and phenol composition, but lacking in TDS content (PW 0) was also evaluated as a reference.

All components were added into deionized and purified water (DI), equivalent to Type II water, and the mixture was sonicated at room temperature for 10 min to enhance solubilization and mixing. The composition of the resulting synthetic solutions, including PW 1000, PW 10,000, and PW 100,000 with the respective NaCl concentrations, is listed in Table 1. These solutions were then subjected to TiO_2_ photocatalytic oxidation preceded by membrane-based pre-treatment, i.e., MD or MF.

### 3.2. Membrane Based Pre-Treatments

#### 3.2.1. Membrane Distillation Setup and Protocol

MD experiments were performed in direct contact configuration, using the bench-scale system described in detail in our previous publications [7,54,55]. The membrane-housing cell consisted of a two-plate frame module measuring 16.5 cm in length, 21.3 cm in width, and 0.19 cm in depth, providing a total active area of 140 cm^2^ (Sterlitech Inc., Auburn, WA, USA). All tests were performed with a commercially available hydrophobic polytetrafluoroethylene (PTFE) membrane (Aquastill, Sittard, The Netherlands), characterized by a thickness of 77 μm, mean pore size of 0.17 μm, porosity of 0.83, and an average water contact angle of 127°. The feed and the distillate streams were circulated in a counter-current mode using two centrifugal pumps. For each stream, the flow rate was kept at 35 L h^−1^ and monitored with two flowmeters (ASA, Sesto San Giovanni, Italy). Initial volumes of 2 L and 1 L were used for the feed and distillate streams, respectively, with the former consisting of the synthetic PW and the latter of DI water. The flux across the membrane was computed by continuously recording the change in weight of the distillate tank over time, through a computer-interfaced scale. The end point was set based on a recovery target of 50% in all tests, corresponding to a concentration factor of 2.

The temperature of the feed stream and of the distillate stream at the inlets of the module were kept at 50° and 25 °C, respectively, adjusting the bulk feed and distillate temperatures by means of a thermostatic bath and of a chiller. To maintain consistency, temperatures were continuously monitored with four thermocouples, which were placed at the inlets and outlets of the membrane module and controlled via an Arduino microcontroller.

#### 3.2.2. Microfiltration Setup and Protocol

Microfiltration (MF) experiments were performed with a cross-flow bench-scale system equipped with an inverter-controlled volumetric pump, a thermally insulated feed tank, and a tubular membrane-housing module [56,57]. A TiO_2_-based tubular ceramic membrane was used, with a molecular weight cut-off of 150 kDa, an inner diameter of 6 mm, a length of 250 mm, and an active area of 47.1 cm^2^ (TAMI Industries, Nyons, France). The filtration tests were conducted with an initial feed volume of 2 L. The cross-flow velocity and the transmembrane pressure were set at 2.4 m s^−1^ and 1 bar, respectively. A tank for the collection of the permeate stream was placed on a computer-interfaced balance; permeate flux was measured continuously by monitoring the change in weight of the permeate solution. The process was carried out at ambient temperature (25 ± 1 °C). The end point was set based on a recovery target of 50% in all tests, corresponding to a concentration factor of 2.

A three-step membrane cleaning was performed after all tests, to ensure uniform membrane characteristics at the beginning of each filtration: (i) repetitive quick flushing with DI water to rinse the filtration unit and remove the remaining PW; (ii) 5 h of operation in backwash mode at a transmembrane pressure difference of 2 bar, using a solution containing NaOH (pH 10) to remove potential humic acid residuals; (iii) 1 h of continuous operation in standard mode with DI water as feed solution, to remove any cleaning agent residues. Note that this cleaning procedure does not represent a standard operational cleaning protocol, but it was specifically designed based on previous studies to restore the initial membrane conditions for research purposes. Its only purpose was to ensure a fair comparison between tests conducted under different conditions [57,58,59].

### 3.3. Photocatalytic Decontamination of Membrane-Treated PW

For photocatalysis experiments, loadings of 0.5 g L^−1^ of TiO_2_ (Ewonik P25) were added into the membrane permeates and distillates, respectively, and placed in cylindrical Pyrex glass cells (4 cm diameter, 2.5 cm height), each containing 5 mL suspension during irradiation. The titania powder was purified from organic and ionic impurities by overnight ultraviolet (UV) irradiation of a water suspension, followed by several washing steps with ultrapure water. The powder was then dried at 80 °C for two hours and gently crushed in a ceramic mortar.

Each irradiation cell was provided with a lateral neck for liquid insertion and withdrawal, and it was tightly closed with a screw cap during irradiation that took place under magnetic stirring. Cells were placed under a UV-A lamp (Philips TL K05, emission maximum at 365 nm), providing an irradiance of 18.8 ± 1.4 W m^−2^ over the illuminated suspensions. In all cases, the lamp was pre-heated for at least 30 min before starting the irradiation experiments. At scheduled time intervals, the cells were withdrawn from the lamp, the suspension was filtered (pore size 0.45 μm, cellulose acetate membranes, Merck Millipore, Burlington, MA, USA) and stored at 4 °C until characterization (vide infra). Control experiments were carried out by dissolving phenol or humic acids in ultra-pure (Milli-Q quality) water.

### 3.4. Analytical Methods

The total organic carbon (TOC) was measured using a TOC-L analyzer (TOC-LDSH FA, E200, Shimadzu, Kyoto, Japan). Calibration was performed using standards of potassium hydrogen phthalate and of NaHCO_3_/Na_2_CO_3_. Additionally, phenol was selectively monitored through a high-performance liquid chromatography system (HPLC) purchased from Shimadzu, which was equipped with a UV–Vis photodiode array detector (SPD-M40), a system controller (SCL-40), a dual solvent pump (LC-40D), an injection valve (DGU-405), an on-line solvent degasser (DGU-14A), a column oven (CTO-40C), and an autosampler (SIL-40). The injection volume was set at 10 μL. A reverse-phase C18 column (ROC C18, 150 m × 4.6 mm, pore size 100 Å, 5 μm packing, gradient grade RESTEK) was used at 40 °C. The isocratic elution was carried out with 70% phosphoric acid (10 mM, pH 2.8) and 30% acetonitrile (gradient grade), at a flow rate of 1 mL min^−1^. Specifically, phenol detection was performed at 220 nm wavelength, and the phenol concentration was obtained from a calibration curve prepared with standard solutions (explored range of 0–3 mg L^−1^ phenol, R^2^ = 0.99, quantification limit 0.03 mg L^−1^, detection limit 0.01 mg L^−1^).

## 4. Conclusions

Membrane treatments were quite effective in removing humic substances from artificial produced water (PW), and MD was also able to achieve a complete removal of NaCl. However, the performance of membrane processes towards the removal of toxic phenol was rather poor. Therefore, although MD performed better than MF towards PW treatment, in either case the resulting permeate stream would not be suitable for either discharge or reuse, due to the presence of residual toxic species.

In this framework, the coupling between membrane pre-treatment and TiO_2_ heterogeneous photocatalysis, applied to the membrane permeate, proved particularly useful. The membrane step removed interfering agents, the absence of which in the permeate was highly helpful to achieve effective photocatalytic degradation of phenol. On the other hand, raw PW proved to be too complex a matrix for a direct photocatalytic treatment to be effective.

Pre-treatment by MD was highly effective because it removed both humic acids and chloride, which interfere with the photocatalytic degradation of phenol. Evidence suggests that irradiated TiO_2_, while very effective in degrading phenol, was not equally able to achieve mineralization of humic acids. However, humic acids are not contaminants sensu stricto and their total removal from PW is not mandatory, unless the water matrix has to undergo additional processes such as chlorination that can produce disinfection by-products [8].

Overall, the aqueous phase resulting from MD pre-treatment plus TiO_2_-based photocatalysis would be suitable for discharge/reuse, due to the absence of salts and toxic phenol and due to the low TOC values (below 1 mg L^−1^). Therefore, the two techniques appear to be complementary to achieve the target of PW decontamination.

## Figures and Tables

**Table 1:** Composition of the synthetic produced waters compared with reference, real streams.

		Synthetic PW					Reference PW
		PW 0	PW 1000	PW 10,000	PW 100,000		
Parameter	Component	Concentration (mg L^−1^)				TOC eq. (mg L^−1^)	Concentration (mg L^−1^)
TOC	Humic Acids	230	230	230	230	60	
	Phenol	2.5	2.5	2.5	2.5	1.9	
	Total organics	232.5	232.5	232.5	232.5	61.9	Maximum~500 [52]
TDS	NaCl	0	1000	10,000	100,000	0	38,500–238,000 [53]

## Data Availability

The original contributions presented in this study are included in the article. Further inquiries can be directed to the corresponding author(s).

## References

[1] Statista. https://www.statista.com/statistics/265229/global-oil-production-in-million-metric-tons/.

[2] Igunnu E.T., Chen G.Z. (2014). Produced Water Treatment Technologies. Int. J. Low-Carbon Technol..

[3] Jiménez S., Andreozzi M., Micó M.M., Álvarez M.G., Contreras S. (2019). Produced Water Treatment by Advanced Oxidation Processes. Sci. Total Environ..

[4] Jiménez S., Micó M.M., Arnaldos M., Medina F., Contreras S. (2018). State of the Art of Produced Water Treatment. Chemosphere.

[5] Al-Ghouti M.A., Al-Kaabi M.A., Ashfaq M.Y., Da’na D.A. (2019). Produced Water Characteristics, Treatment and Reuse: A Review. J. Water Process Eng..

[6] Ganiyu S.O., Sable S., Gamal El-Din M. (2022). Advanced Oxidation Processes for the Degradation of Dissolved Organics in Produced Water: A Review of Process Performance, Degradation Kinetics and Pathway. Chem. Eng. J..

[7] Ricceri F., Giagnorio M., Farinelli G., Blandini G., Minella M., Vione D., Tiraferri A. (2019). Desalination of Produced Water by Membrane Distillation: Effect of the Feed Components and of a Pre-Treatment by Fenton Oxidation. Sci. Rep..

[8] Sciscenko I., Vione D., Minella M. (2024). Infancy of Peracetic Acid Activation by Iron, a New Fenton-Based Process: A Review. Heliyon.

[9] Nosaka Y., Nosaka A.Y. (2017). Generation and Detection of Reactive Oxygen Species in Photocatalysis. Chem. Rev..

[10] Malato S., Fernández-Ibáñez P., Maldonado M.I., Blanco J., Gernjak W. (2009). Decontamination and Disinfection of Water by Solar Photocatalysis: Recent Overview and Trends. Catal. Today.

[11] Sordello F., Calza P., Minero C., Malato S., Minella M. (2022). More than One Century of History for Photocatalysis, from Past, Present and Future Perspectives. Catalysts.

[12] Dar M.I., Chandiran A.K., Grätzel M., Nazeeruddin M.K., Shivashankar S.A. (2014). Controlled Synthesis of TiO_2_ Nanoparticles and Nanospheres Using a Microwave Assisted Approach for Their Application in Dye-Sensitized Solar Cells. J. Mater. Chem. A.

[13] Syed M.A., Mauriya A.K., Shaik F. (2022). Investigation of Epoxy Resin/Nano-TiO_2_ Composites in Photocatalytic Degradation of Organics Present in Oil-Produced Water. Int. J. Environ. Anal. Chem..

[14] Andreozzi M., Álvarez M.G., Contreras S., Medina F., Clarizia L., Vitiello G., Llorca J., Marotta R. (2018). Treatment of Saline Produced Water through Photocatalysis Using rGO-TiO_2_ Nanocomposites. Catal. Today.

[15] Chen L., Xu P., Wang H. (2022). Photocatalytic Membrane Reactors for Produced Water Treatment and Reuse: Fundamentals, Affecting Factors, Rational Design, and Evaluation Metrics. J. Hazard. Mater..

[16] da Fonseca Ferreira A.D., Coelho D.R.B., dos Santos R.V.G., Nascimento K.S., de Andrade Presciliano F., da Silva F.P., Campos J.C., da Fonseca F.V., Borges C.P., Weschenfelder S.E. (2021). Fouling Mitigation in Produced Water Treatment by Conjugation of Advanced Oxidation Process and Microfiltration. Environ. Sci. Pollut. Res..

[17] Coha M., Farinelli G., Tiraferri A., Minella M., Vione D. (2021). Advanced Oxidation Processes in the Removal of Organic Substances from Produced Water: Potential, Configurations, and Research Needs. Chem. Eng. J..

[18] Conrad C.L., Ben Yin Y., Hanna T., Atkinson A.J., Alvarez P.J.J., Tekavec T.N., Reynolds M.A., Wong M.S. (2020). Fit-for-Purpose Treatment Goals for Produced Waters in Shale Oil and Gas Fields. Water Res..

[19] Al-Rasheed R., Cardin D.J. (2003). Photocatalytic Degradation of Humic Acid in Saline Waters. Part 1. Artificial Seawater: Influence of TiO_2_, Temperature, pH, and Air-Flow. Chemosphere.

[20] Krivec M., Dillert R., Bahnemann D.W., Mehle A., Štrancar J., Dražić G. (2014). The Nature of Chlorine-Inhibition of Photocatalytic Degradation of Dichloroacetic Acid in a TiO_2_-Based Microreactor. Phys. Chem. Chem. Phys..

[21] Chen H.Y., Zahraa O., Bouchy M. (1997). Inhibition of the Adsorption and Photocatalytic Degradation of an Organic Contaminant in an Aqueous Suspension of TiO_2_ by Inorganic Ions. J. Photochem. Photobiol. A Chem..

[22] Minella M., De Bellis N., Gallo A., Giagnorio M., Minero C., Bertinetti S., Sethi R., Tiraferri A., Vione D. (2018). Coupling of Nanofiltration and Thermal Fenton Reaction for the Abatement of Carbamazepine in Wastewater. ACS Omega.

[23] Vesterkvist P.S.M., Misiorek J.O., Spoof L.E.M., Toivola D.M., Meriluoto J.A.O. (2012). Comparative Cellular Toxicity of Hydrophilic and Hydrophobic Microcystins on Caco-2 Cells. Toxins.

[24] Farinelli G., Coha M., Minella M., Fabbri D., Pazzi M., Vione D., Tiraferri A. (2021). Evaluation of Fenton and Modified Fenton Oxidation Coupled with Membrane Distillation for Produced Water Treatment: Benefits, Challenges, and Effluent Toxicity. Sci. Total Environ..

[25] Horseman T., Yin Y., Christie K.S., Wang Z., Tong T., Lin S. (2021). Wetting, Scaling, and Fouling in Membrane Distillation: State-of-the-Art Insights on Fundamental Mechanisms and Mitigation Strategies. ACS EST Eng..

[26] Pasternak G., Kołwzan B. (2013). Surface Tension and Toxicity Changes during Biodegradation of Carbazole by Newly Isolated Methylotrophic Strain *Methylobacterium* Sp. GPE1. Int. Biodeterior. Biodegrad..

[27] Nghiem L.D., Cath T. (2011). A Scaling Mitigation Approach during Direct Contact Membrane Distillation. Sep. Purif. Technol..

[28] Rezaei M., Warsinger D.M., Lienhard V.J.H., Duke M.C., Matsuura T., Samhaber W.M. (2018). Wetting Phenomena in Membrane Distillation: Mechanisms, Reversal, and Prevention. Water Res..

[29] Eziyi I., Krothapalli A., Osorio J.D., Ordonez J.C., Vargas J.V.C. Effects of Salinity and Feed Temperature on Permeate Flux of an Air Gap Membrane Distillation Unit for Sea Water Desalination. Proceedings of the 2013 1st IEEE Conference on Technologies for Sustainability (SusTech).

[30] Warsinger D.M., Swaminathan J., Guillen-Burrieza E., Arafat H.A., Lienhard V.J.H. (2015). Scaling and Fouling in Membrane Distillation for Desalination Applications: A Review. Desalination.

[31] Yuan W., Zydney A.L. (1999). Humic Acid Fouling during Microfiltration. J. Membr. Sci..

[32] Yu Y., Yang Z., Duan Y. (2017). Structure and Flow Calculation of Cake Layer on Microfiltration Membranes. J. Environ. Sci..

[33] Yuan W., Kocic A., Zydney A.L. (2002). Analysis of Humic Acid Fouling during Microfiltration Using a Pore Blockage–Cake Filtration Model. J. Membr. Sci..

[34] McGaughey A.L., Childress A.E. (2022). Wetting Indicators, Modes, and Trade-Offs in Membrane Distillation. J. Membr. Sci..

[35] Song M., Song B., Meng F., Chen D., Sun F., Wei Y. (2019). Incorporation of Humic Acid into Biomass Derived Carbon for Enhanced Adsorption of Phenol. Sci. Rep..

[36] Minero C., Mariella G., Maurino V., Vione D., Pelizzetti E. (2000). Photocatalytic Transformation of Organic Compounds in the Presence of Inorganic Ions. 2. Competitive Reactions of Phenol and Alcohols on a Titanium Dioxide−Fluoride System. Langmuir.

[37] Li Y., Nie W., Liu Y., Huang D., Xu Z., Peng X., George C., Yan C., Tham Y.J., Yu C. (2020). Photoinduced Production of Chlorine Molecules from Titanium Dioxide Surfaces Containing Chloride. Environ. Sci. Technol. Lett..

[38] Neta P., Huie R.E., Ross A.B. (1988). Rate Constants for Reactions of Inorganic Radicals in Aqueous Solution. J. Phys. Chem. Ref. Data.

[39] Wojnárovits L., Wang J., Chu L., Takács E. (2022). Rate Constants of Chlorine Atom Reactions with Organic Molecules in Aqueous Solutions, an Overview. Environ. Sci. Pollut. Res..

[40] Buxton G.V., Greenstock C.L., Helman W.P., Ross A.B. (1988). Critical Review of Rate Constants for Reactions of Hydrated Electrons, Hydrogen Atoms and Hydroxyl Radicals (^•^OH/^•^O^−^ in Aqueous Solution. J. Phys. Chem. Ref. Data.

[41] Maurino V., Minella M., Sordello F., Minero C. (2016). A Proof of the Direct Hole Transfer in Photocatalysis: The Case Ofmelamine. Appl. Catal. A Gen..

[42] Chen D., Cheng Y., Zhou N., Chen P., Wang Y., Li K., Huo S., Cheng P., Peng P., Zhang R. (2020). Photocatalytic Degradation of Organic Pollutants Using TiO_2_-Based Photocatalysts: A Review. J. Clean. Prod..

[43] Uyguner C.S., Bekbolet M. (2010). TiO_2_-Assisted Photocatalytic Degradation of Humic Acids: Effect of Copper Ions. Water Sci. Technol..

[44] Baalousha M., Motelica-Heino M., Coustumer P.L. (2006). Conformation and Size of Humic Substances: Effects of Major Cation Concentration and Type, pH, Salinity, and Residence Time. Colloids Surf. A Physicochem. Eng. Asp..

[45] Lin L., Jiang W., Chen L., Xu P., Wang H. (2020). Treatment of Produced Water with Photocatalysis: Recent Advances, Affecting Factors and Future Research Prospects. Catalysts.

[46] Dharma H.N.C., Jaafar J., Widiastuti N., Matsuyama H., Rajabsadeh S., Othman M.H.D., Rahman M.A., Jafri N.N.M., Suhaimin N.S., Nasir A.M. (2022). A Review of Titanium Dioxide (TiO_2_)-Based Photocatalyst for Oilfield-Produced Water Treatment. Membranes.

[47] Valadez-Renteria E., Barrera-Rendon E., Oliva J., Rodriguez-Gonzalez V. (2021). Flexible CuS/TiO_2_ Based Composites Made with Recycled Bags and Polystyrene for the Efficient Removal of the 4-CP Pesticide from Drinking Water. Sep. Purif. Technol..

[48] Andrade M.A., Carmona R.J., Mestre A.S., Matos J., Carvalho A.P., Ania C.O. (2014). Visible Light Driven Photooxidation of Phenol on TiO_2_/Cu-Loaded Carbon Catalysts. Carbon.

[49] Vasantharaj S., Sathiyavimal S., Senthilkumar P., Kalpana V.N., Rajalakshmi G., Alsehli M., Elfasakhany A., Pugazhendhi A. (2021). Enhanced Photocatalytic Degradation of Water Pollutants Using Bio-Green Synthesis of Zinc Oxide Nanoparticles (ZnO NPs). J. Environ. Chem. Eng..

[50] Lakshmi K., Kadirvelu K., Mohan P.S. (2019). Chemically Modified Electrospun Nanofiber for High Adsorption and Effective Photocatalytic Decontamination of Organophosphorus Compounds. J. Chem. Technol. Biotechnol..

[51] Shanaah H.H., Alzaimoor E.F.H., Rashdan S., Abdalhafith A.A., Kamel A.H. (2023). Photocatalytic Degradation and Adsorptive Removal of Emerging Organic Pesticides Using Metal Oxide and Their Composites: Recent Trends and Future Perspectives. Sustainability.

[52] Estrada J.M., Bhamidimarri R. (2016). A Review of the Issues and Treatment Options for Wastewater from Shale Gas Extraction by Hydraulic Fracturing. Fuel.

[53] Bertozzi E., Craveri L., Malaguti M., Ricceri F., Carone M., Riggio V., Tiraferri A. (2024). Concentration of Phycocyanin and Coffee Extracts in Aqueous Solutions with Osmotically-Assisted Membrane Distillation. Sep. Purif. Technol..

[54] Sampling and Analysis of Water Streams Associated with the Development of Marcellus Shale Gas—AmeriGEOSS Community Platform DataHub. (BETA). https://data.amerigeoss.org/tr/dataset/sampling-and-analysis-of-water-streams-associated-with-the-development-of-marcellus-shale-gas.

[55] Morciano M., Malaguti M., Ricceri F., Tiraferri A., Fasano M. (2024). Process Optimization of Osmotic Membrane Distillation for the Extraction of Valuable Resources from Water Streams. npj Clean Water.

[56] Ricceri F., Malaguti M., Derossi C., Zanetti M., Riggio V., Tiraferri A. (2022). Microalgae Biomass Concentration and Reuse of Water as New Cultivation Medium Using Ceramic Membrane Filtration. Chemosphere.

[57] Lowe J., Hossain M.M. (2008). Application of Ultrafiltration Membranes for Removal of Humic Acid from Drinking Water. Desalination.

[58] Malaguti M., Craveri L., Ricceri F., Riggio V., Zanetti M., Tiraferri A. (2023). Dewatering of *Scenedesmus obliquus* Cultivation Substrate with Microfiltration: Potential and Challenges for Water Reuse and Effective Harvesting. Engineering.

[59] Malaguti M., Novoa A.F., Ricceri F., Giagnorio M., Vrouwenvelder J.S., Tiraferri A., Fortunato L. (2022). Control Strategies against Algal Fouling in Membrane Processes Applied for Microalgae Biomass Harvesting. J. Water Process Eng..

